# Manufacturing and banking canine adipose-derived mesenchymal stem cells for veterinary clinical application

**DOI:** 10.1186/s12917-021-02791-3

**Published:** 2021-03-01

**Authors:** Huina Luo, Dongsheng Li, Zhisheng Chen, Bingyun Wang, Shengfeng Chen

**Affiliations:** 1 School of Life Science and Engineering, Foshan University, 528231 Foshan, Guangdong China; 2 VetCell Biotechnology Company Limited, 528231 Foshan, Guangdong China

**Keywords:** Canine, Cell therapy, Mesenchymal stem cells, Adipose, Banking

## Abstract

**Background:**

Mesenchymal stem cells (MSCs) have generated a great amount of interest in recent years as a novel therapeutic application for improving the quality of pet life and helping them free from painful conditions and diseases. It has now become critical to address the challenges related to the safety and efficacy of MSCs expanded in vitro. In this study, we establish a standardized process for manufacture of canine adipose-derived MSCs (AD-MSCs), including tissue sourcing, cell isolation and culture, cryopreservation, thawing and expansion, quality control and testing, and evaluate the safety and efficacy of those cells for clinical applications.

**Results:**

After expansion, the viability of AD-MSCs manufactured under our standardized process was above 90 %. Expression of surface markers and differentiation potential was consistent with ISCT standards. Sterility, mycoplasma, and endotoxin tests were consistently negative. AD-MSCs presented normal karyotype, and did not form in vivo tumors. No adverse events were noted in the case treated with intravenously AD-MSCs.

**Conclusions:**

Herein we demonstrated the establishment of a feasible bioprocess for manufacturing and banking canine AD-MSCs for veterinary clinical use.

## Background

Mesenchymal stem cells (MSCs) which were first discovered by Friedenstein from bone marrow derived cells are a type of self-renewing cells harboring the potential to differentiate into diverse tissues, such as bone, cartilage, muscle, fat and tendon [1–5]. At present, in human clinical medicine, a variety of clinical trials with human MSCs are ongoing for the treatment of immunological diseases and degenerative diseases [6–9]. Currently, it has been reported that MSCs could be isolated from various tissues other than bone marrow [10], such as adipose [11], umbilical cord [12], dental pulp [13], placenta [14] and amniotic membrane [15]. Adipose-derived Mesenchymal stem cells (AD-MSCs) have gained in popularity due to the ease and abundance of harvesting adipose tissue, greater capacity of self-renewal multipotency and paracrine immunomodulatory [11, 16–18].

As one of the most common companion animals, some breeds of canine including beagles are also experimental animals. With the improvement of living standards, diseases of aging in canine have become more common clinically, especially chronic diseases, such as osteoarthritis, hip dysplasia, diabetes, pancreatitis and tendonopathy [19, 20]. Meanwhile, there are no particularly effective treatments for these diseases, which not only affect the quality of life of the affected animals, but also require the owners to invest a lot of energy, time and money. Currently, many related reports have confirmed that AD-MSCs therapy in a variety of canine diseases in clinical trials have shown initial results [21–25].

As one kind of superior cytotherapy, canine AD-MSCs therapy has great clinical application potential in various chronic diseases treatment [26, 27]. Although the technology of canine AD-MSCs isolation and expansion at laboratory scale is relatively mature, manufacturing a therapeutic cell-based product is and will continue to be challenging [28, 29]. In this paper, we established a standardized operating procedure for manufacturing canine AD-MSCs, which encompasses tissue sourcing, cell isolation and culture, cryopreservation, thawing and expansion, quality control and testing, and evaluate the safety and efficacy of those cells for clinical applications. The establishment of canine AD-MSCs bank was expected to provide an adequate source of seed cells for the cell therapy of canine diseases and the research of tissue engineering products.

## Result

### Cell isolation,culture and cryopreservation

**Fig. 1:**
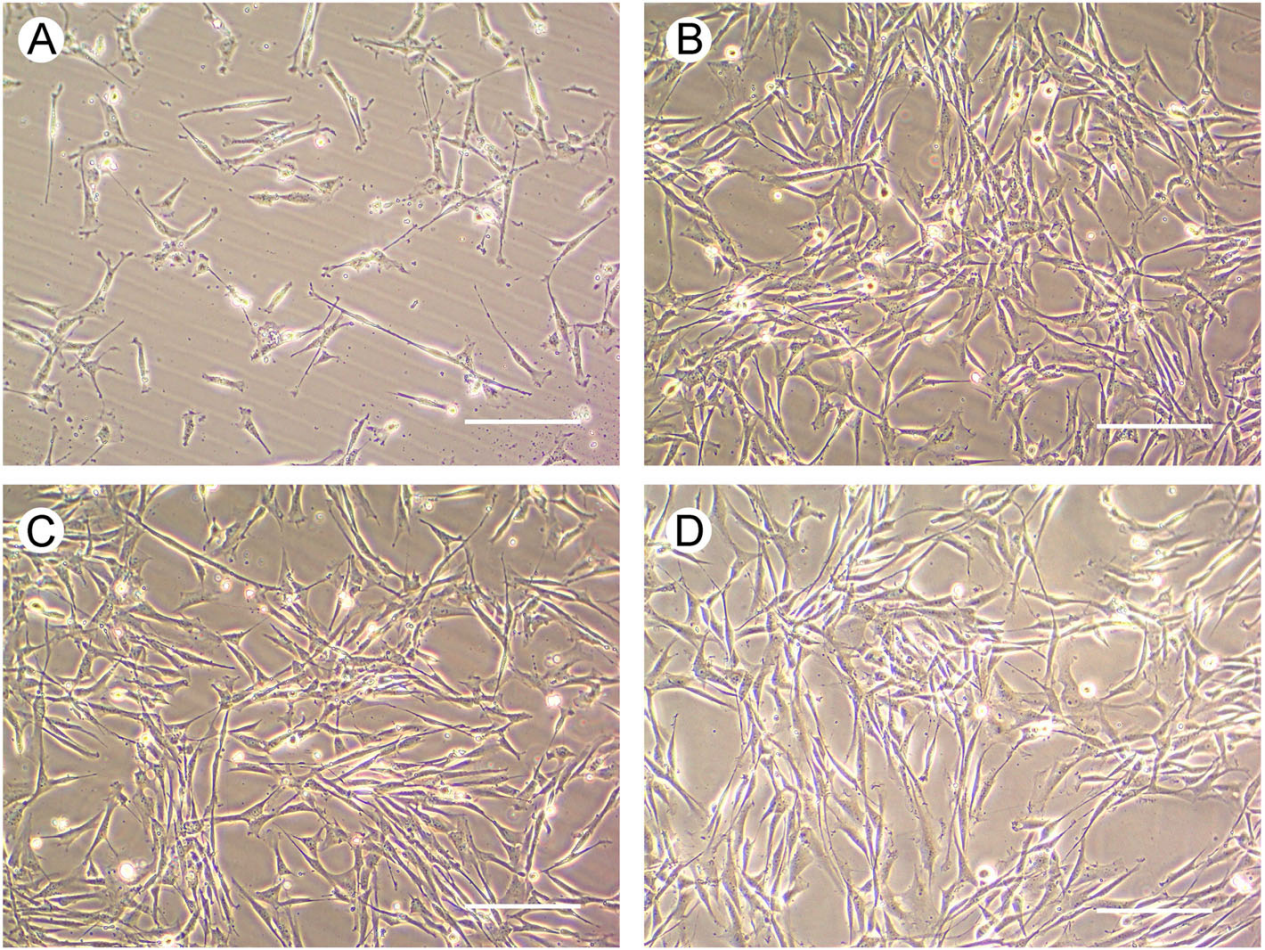
Morphological observations of canine AD-MSCs in different generations. **a** Canine AD-MSCs on day 4 of primary culture. Adherent spindle cells appeared and most of the other suspension cells died and disappeared; (**b** and **c**) Morphology of canine AD-MSCs in *passage 1 and passage 2*, respectively. Cells were very pure and homogeneous with a typical long spindle-shape. **d** Morphology of thawed canine AD-MSCs in *passage 4*. Cell morphology and proliferation ability were maintained after cryopreservation. Scale bar, 200 µm

Approximately 2–6 × 10^6^ nucleated cells were obtained from adipose tissue (*n* = 12, range 2–5 grams) after collagenase type I digestion and seeded into 1–2 100mm cell -culture dishes. After 48 h of growth, the vast majority of AD-MSCs were attached to the culture plate and exhibited fibroblast-like morphology (Fig. 1). The cells grew to 80 % − 90 % confluence after 5–7 days and were passaged for the first time. After second passages with a 1:3 split ratio, 0.64–2.67 × 10^7^ AD-MSC*s passage 2* were harvested and cryopreserved at the end of first expansion period. Each batch of AD-MSCs must passed the in-process test and be given a unique identifier before cryopreservation. Viability of the freshly harvested cells was greater than 90 % in all cases. Basic data for the MSC expansions are shown in Table 1.

**Table 1:** The number of harvested cells in different passages

No.	Size(g)	Harvested Cells of P0 (10^6^)	Harvested Cells of P1 (10^6^)	Harvested Cells of P2 (10^6^)
CAD-001	3.2	3.7	10.5	26.7
CAD-002	4.1	4.2	12.3	24.3
CAD-003	2.1	2.2	4.1	7.8
CAD-004	2.4	4.3	8.3	17.3
CAD-005	3.2	3.2	4.7	9.3
CAD-006	3.2	4.1	9.2	10.4
CAD-007	4.5	4.7	12.2	26.7
CAD-008	1.2	4.3	8.9	19.2
CAD-009	1.9	3.0	6.4	12.6
CAD-010	2.4	4.1	9.4	19.9
CAD-011	6.1	5.8	10.7	23.5
CAD-012	3.1	5.9	12.7	25.9

### Characterization and quality controls

AD-MSCs presented subsequently fibrous or fusiform shape and showed fibroblast-like adherent growth (Fig. 1).

**Fig. 2:**
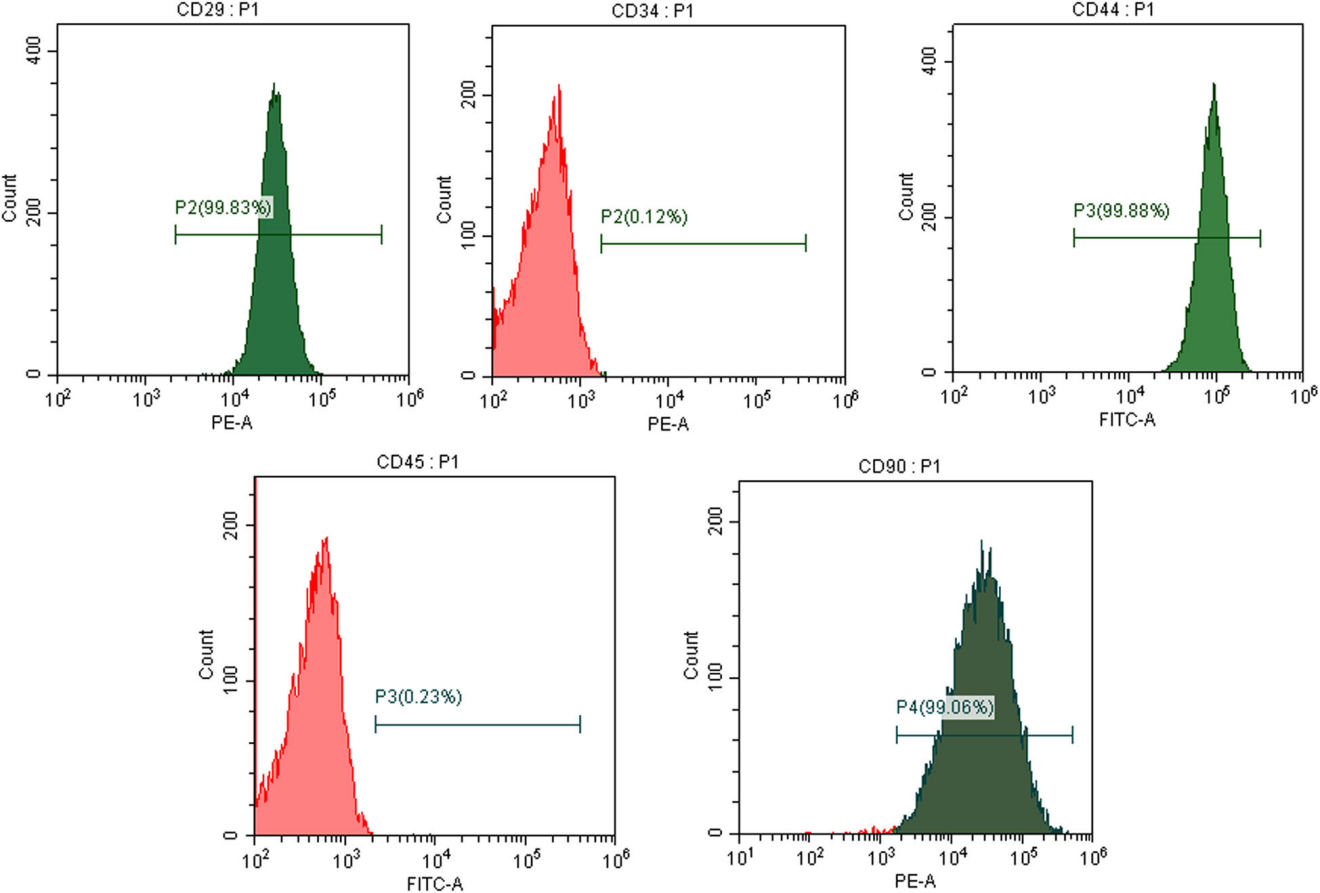
Surface markers of canine AD-MSCs in *passage 2*. Based on flow cytometric analysis, surface molecule markersCD29, CD44 and CD90 were highly expressed (> 95 %) on canine AD-MSCs in *passage 2 and passage 5*, whereas the expression of hematopoietic stem cell markers CD34 and leukocyte common antigen CD45 < 2 %

The Cells of *passage 2* and *passage 5* were highly-expressed mesenchymal stem cell surface markers CD29, CD44 and CD90, while for the lowly-expressed haematopoietic stem cells surface markers CD34 and leukocyte common antigen CD45. The positive expression rate for CD29, CD44 and CD90, in was > 95 %, and the positive expression rate for CD34 and CD45 was < 2 % (Table 2; Fig. 2).

**Table 2:** Surface marker expression of AD-MSCs

Surface marker	Expression (%)	
	Passage 2	Passage 5
CD 29	99.35 ± 0.48	99.25 ± 0.59
CD 34	0.18 ± 0.06	0.29 ± 0.31
CD 44	99.22 ± 0.59	98.27 ± 0.73
CD 45	0.47 ± 0.38	0.54 ± 0.28
CD 90	98.32 ± 0.99	98.08 ± 1.43

**Fig. 3:**
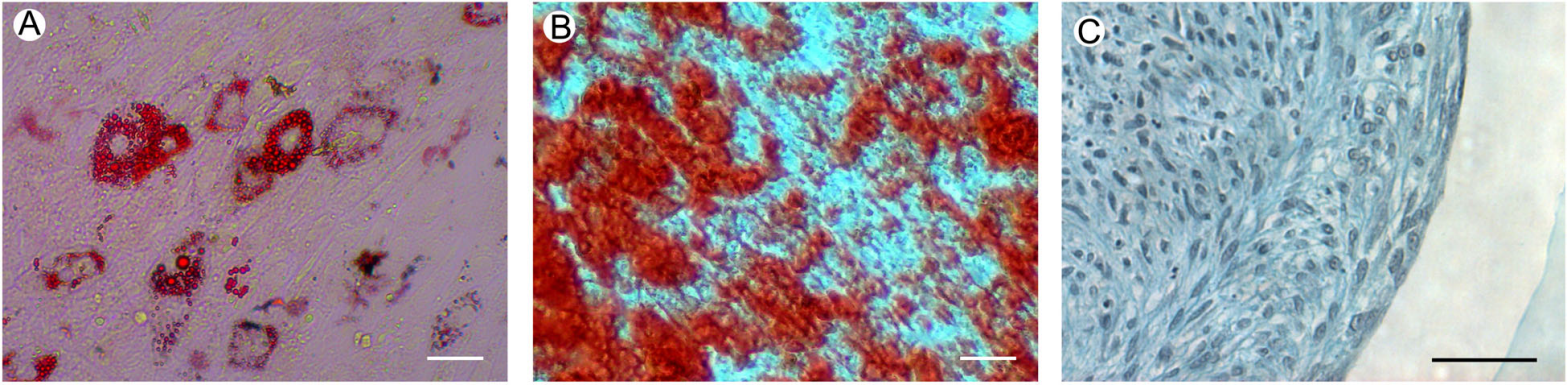
Adipogenic, osteogenic and chondrogenic differentiation of canine AD-MSCs in *passage 2*. **a** Following adipogenic induction for 14 days, canine AD-MSCs were positive for Oil red-O staining and contained an abundance of lipid droplets. **b** Following osteogenic induction for 14 days, canine AD-MSCs in were positive for alizarin red staining. **c** Following chondrogenic induction for 21 days, canine AD-MSCs in were positive for alcian blue staining. Scale bars, 50 µm

The ability of adipogenic, osteogenic and chondrogenic differentiation in vitro was detected respectively by Oil red O, alizarin red and alcian blue staining. The adipogenic differentiated AD-MSCs were visualized by staining with Oil red-O on day 15. Calcium nodules were observed and stained red by alizarin red in osteogenic induction groups on day 17. Representative of chondrogenic differentiation detected by alcian blue staining at day 28 (Fig. 3).

**Fig. 4:**
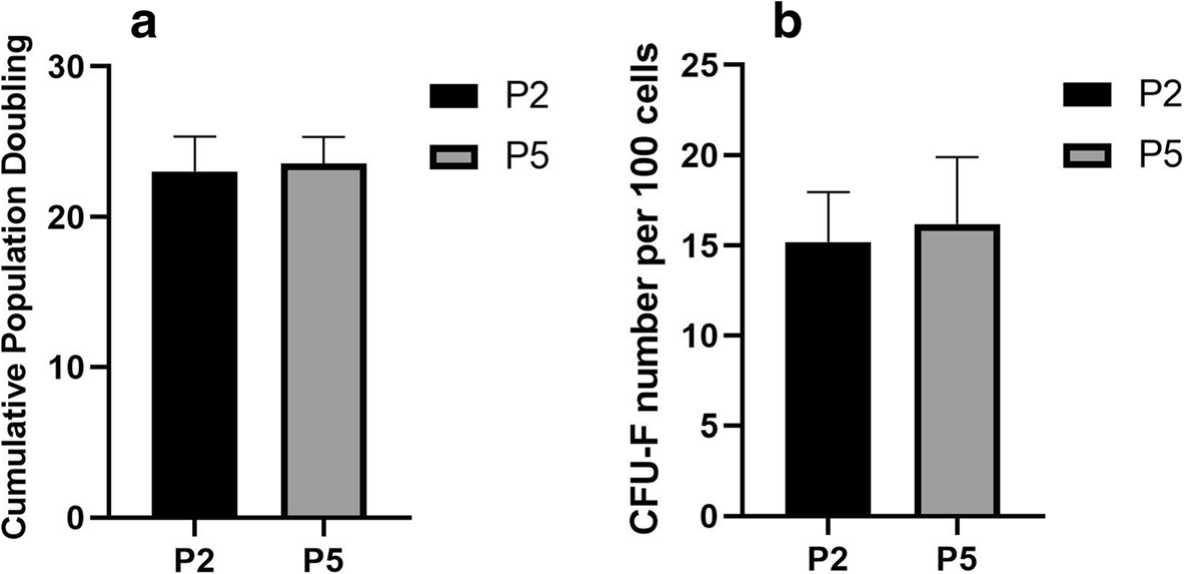
Growth characteristics of canine AD-MSCs in *passage 2 and passage 5*. **a** cumulative population doubling and **b** Colony-Forming unit-fibroblasts capacity in *passage 2 and passage 5*. ^*^P < 0.05. CFU-F, Colony-Forming unit-fibroblasts capacity. P2, *passage 2*; P5, *passage 5*

The population doubling time and Colony-Forming unit-fibroblasts capacity of AD-MSCs was used to assess proliferative ability. The population doubling time was 22.03 ± 2.30 hours in *passage 2* and 23.62 ± 1.67 hours in *passage 5*. The Colony-Forming unit-fibroblasts capacity of AD-MSCs of in *passage 2* and *passage 5* were 15.20 ± 2.77 % and 16.20 ± 3.70 %, respectively (Fig. 4).

Sterility, mycoplasma, and endotoxin test were performed before cryopreservation and release of final product, as well as by random testing of culture supernatants in the course of the expansion. All the pre-cryopreservation cells and final products were free of microbial and mycoplasma contamination. Endotoxin levels of pre-cryopreservation cells and final products were lower than 0.5 EU/ml.

**Fig. 5:**
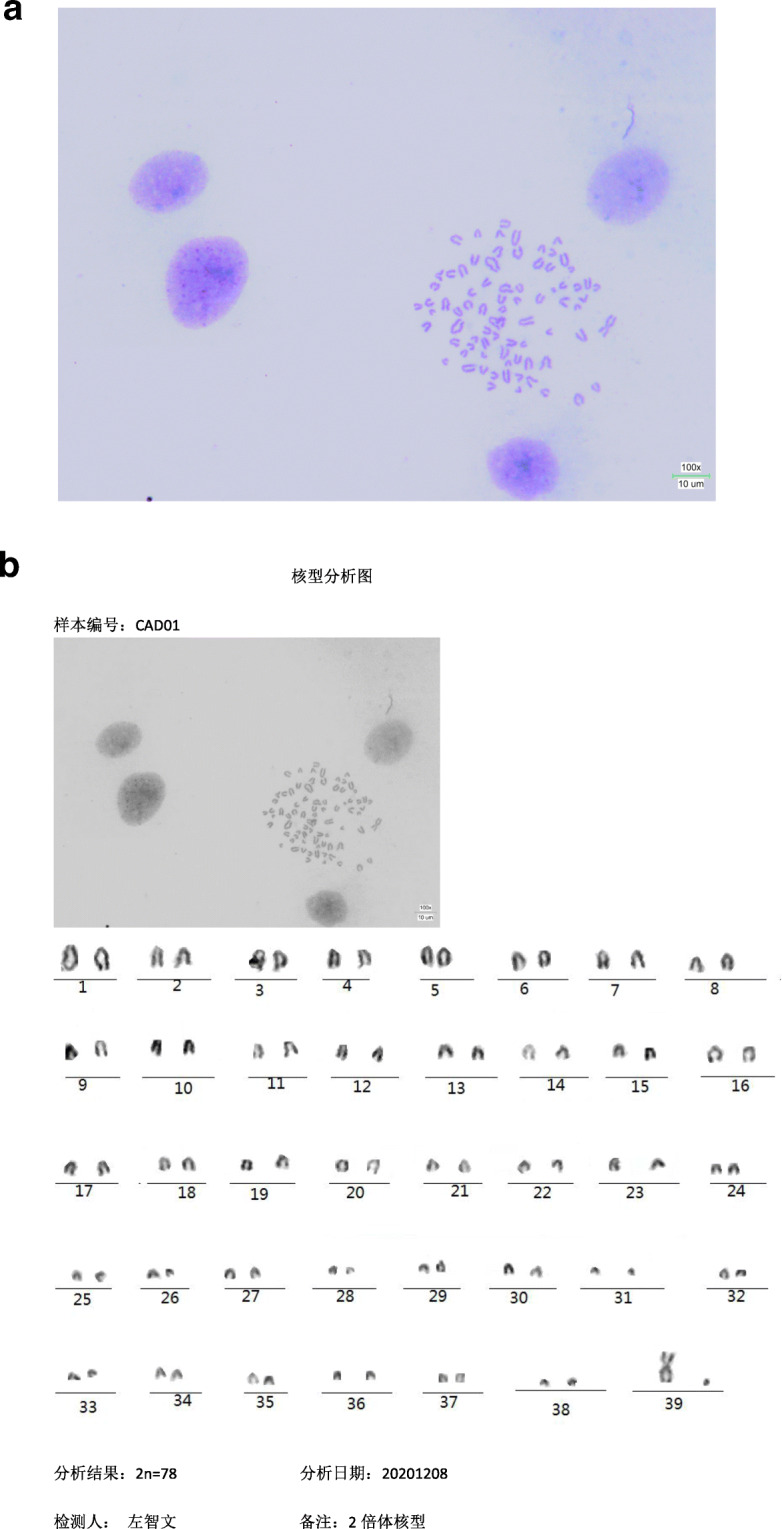
Chromosome and Karyotype analysis of canine AD-MSCs in passage 5. **a** Metaphase chromosome analysis (magnification: ×1,000) and **b** karyotype analysis of AD-MSCs. The number of chromosomes in the AD-MSCs was containing 38 pairs of euchromosomes plus two sex chromosomes (XY)

**Fig. 6:**
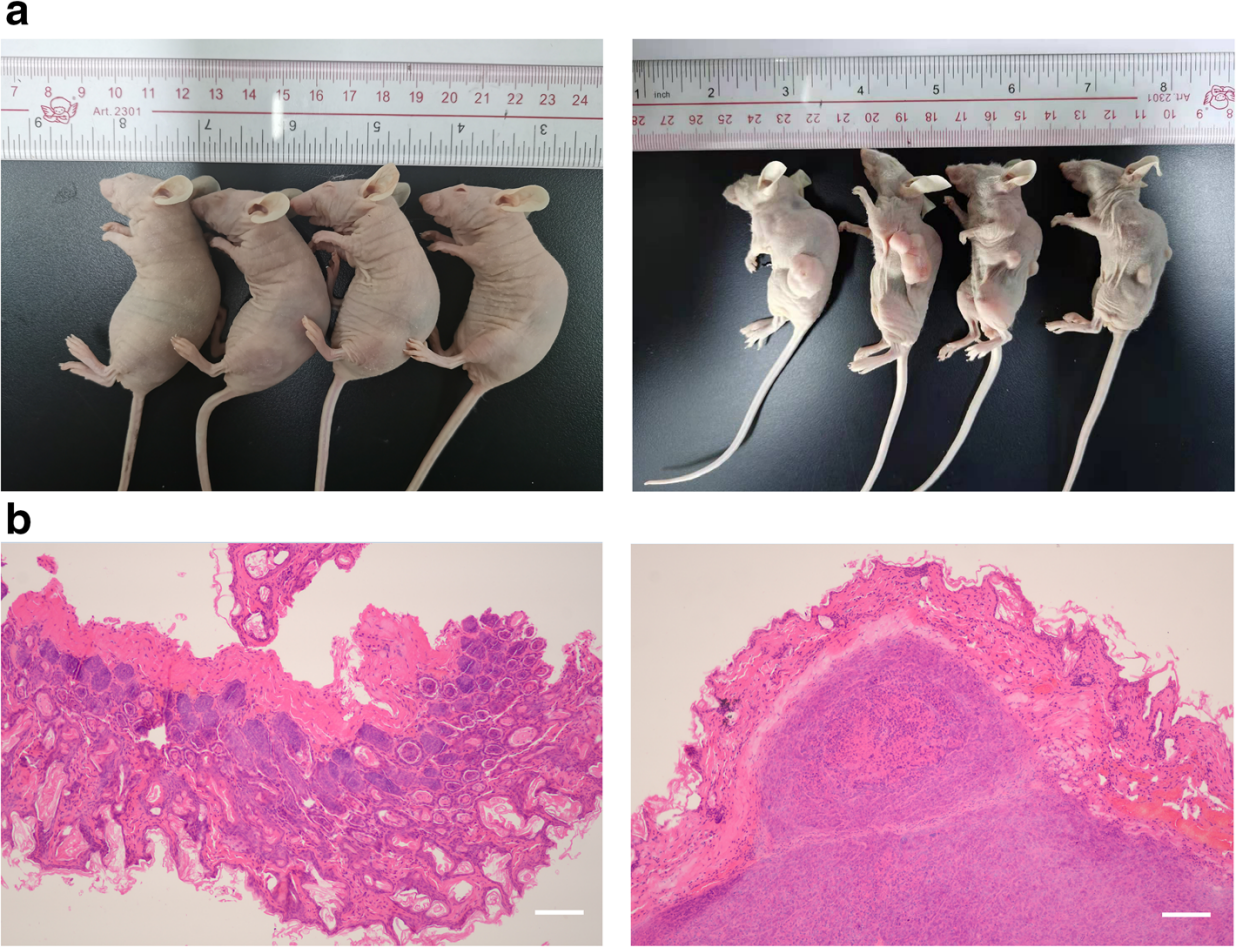
Tumorigenicity evaluation of canine AD-MSCs in passage 5. **a** No tumor formation was observed in the AD-MSCs group within 40 d after subcutaneous injection in mice (arrow: visible tumor in Hela cells group). **b** Histopathological examination of hematoxylin and eosin staining in injection sites. No cell mass formation was observed in the AD-MSCs group. However, the HeLa cells injection site showed a subcutaneous mass. Scale bars, 100 µm

AD-MSCs were diploid, containing 78 chromosomes and no abnormalities were detected (Fig. 5). No evidence of tumor formation of observation in mice and the skin at the site of AD-MSCs injection did not contain residual detectable tumor cells, as noted by histopathology (Fig. 6).

### Cell therapy

The case showed an unexpected response to stem cell therapy. After intravenous transplantation of AD-MSCs, the spirit state of the infected dog improved on the day of treatment, and the number of white blood cells increased on the second day and returned to normal on the third day (Table 3). Control group case had no obvious improvement, and the number of white blood cells were extremely low and neutrophils were reduced to undetectable levels on the second day. And eventually, the control group dog died on the third day. In general, stem cell therapy combined with conventional treatment of canine parvovirus infection has the advantages of rapid cure, short course of treatment, good effect, and no adverse reactions. There were no adverse reactions in response to AD-MSCs therapy.

**Table 3:** Hemogram levels before and after AD-MSCs transplantation treatment

	Prior Treatment	2 d after Treatment	3 d after Treatment	Reference Range
WBC (10^9^/L)	0.9	1.1	7.3	6.0–17.0
Lym (10^9^/L)	0.2	0.4	2.5	5.1–10.8
Mon (10^9^/L)	0	0	0.4	0-1.8
Gran (10^9^/L)	0.7	0.7	4.4	4.0-12.6

*WBC* white blood cell, *Lym *lymphocyte, *Mon *monocyte, *Gran *granulocyte

## Discussion

AD-MSCs are a type of adult stem cell deriving from adipose tissues with self-renewal ability and multidirectional differentiation potential [30]. As an attractive source of MSCs, adipose tissue usually contains far more MSCs than other sources contain [31]. AD-MSCs have become the focus of considerable interest in regenerative tissue engineering due to their easy access and availability in large quantities. The different methods for isolation and culture of canine AD-MSCs has also been established across different countries and different laboratories [22, 24, 26]. Currently, MSCs manufacture including isolation, culture and identification methods are labor intensive, with many time-consuming steps [3, 32]. A complex set of processes introduces the risk of microbial infection and changes in biologic properties [33]. Accordingly, all steps of cell manufacture for clinical application must be performed based on good manufacturing practice to achieve a reasonable safety and quality [34]. The establishment of canine AD-MSCs bank operating in accordance with good manufacturing practice standards will accelerates the clinical application of canine stem cell therapy and the development of stem cell industrialization in pet market.

In this study, we tried to establish a standardized operating process encompasses tissue sourcing, cell isolation and culture, cryopreservation, thawing and expansion for clinical grade canine AD-MSCs manufacturing. Adipose tissue samples were aseptically collected from abdomen fat of healthy donor canine under general anaesthesia. Donor canine peripheral blood was then taken for the examination of infectious diseases including RV, CDV, ICHV, CPV, CCV, fungi and bacteria. Subsequent process can be carried out after the qualification is confirmed, which will serve as the initial basis for the study of cell quality. Using mechanical disruption combined with collagenase type I digestion, AD-MSCs could be successfully isolated and further seeded centrifugally into cell culture dishes in our optimum culture conditions.

After thawing and expansion, AD-MSCs showed all properties characteristic for mesenchymal stem cells outlined by the International Society for Cellular Therapy (ISCT) for defining MSCs [35]. The cells were in a fibrocyte-like form and had a spindle-shape, and were arranged in a whirlpool pattern. The cells formed a monolayer of homogenous bipolar spindle-like cells with a whirl-pool-like array. The immunophenotype analysis displayed that the cells positively expressed of CD29, CD44, and CD90 and the lack of CD34, and CD45. In addition, they demonstrated the ability of osteogenic, chondrogenic and adipogenic differentiation.

The manufacturing of AD-MSCs as cell therapy products for veterinary clinical application should be performed with corresponding controls to ensure its safety and quality [36, 37]. The AD-MSCs have undergone safety testing including sterility, and mycoplasma and results found to meet specifications. There were no bacterial, fungal, or mycoplasma contaminations observed in any of our 12 cultures. The passaged AD-MSCs maintained normal karyotype and had no evidence of tumorigenicity. With a small number of cells and a limited number of injections, the administration of AD-MSCs was demonstrated intravenously without any immediate adverse events.

In comparison with stem cells derived from other sources, AD-MSCs have the advantages of convenient material acquisition, fewer ethical issues, low damage and abundant sources [38, 39]. Our previous study indicated that canine AD-MSCs had the highest proliferation activity vis-à-vis four other sources-derived MSCs in vitro. On average, the number of AD-MSCs doubled every day in our cell culture system [40]. The higher rates of cell proliferation help to harvest more numbers of MSCs in the same unit of time, thus saving cell production costs and times. In addition, the establishment of AD-MSCs banks may accelerate the veterinary clinical application of stem cell therapy and the development of pet stem cell businesses.

## Conclusions

In summary, we successfully established a standardized process for manufacturing and banking canine AD-MSCs for clinical use and confirmed adipose tissues representing an appealing source of MSCs for cell therapy. It is hoped that the clinical experimental research and application of canine AD-MSCs in regenerative medicine will benefit the majority of sick animals and contribute to the health of pet dogs.

## Materials and methods

### Adipose tissue collection

Canine abdominal subcutaneous adipose was collected during routine clinically indicated surgery at Affiliated Animal Hospital of Foshan University from 2017 to 2019. All owners agreed with the collection of tissue and signed an informed consent. A screen of the medical history was performed and a blood sample was tested for specific canine pathogens, such as rabies virus (RV), canine distemper virus (CDV), infectious canine hepatitis virus (ICHV), canine parvovirus (CPV), canine coronaviruses (CCV), fungi and bacteria. Tissue collection was approved by the Animal Ethics Committee of Foshan University, and were conducted in accordance with the ethical standards of the university. Adipose tissue immersed in DMEM (Hyclone, USA) supplemented 1 % penicillin and streptomycin (Gibco, USA) immediately transported to the lab at 4–10℃.

### Cell isolation and culture

MSCs were isolated from adipose tissue based on methods previously described [40]. Briefly, tissue was digested with collagenase type I. Then filtration through a 100-mesh cell strain, the filtrate was centrifuged to collect AD-MSCs. Approximately 5000 isolated suspended cells per cm^2^ were transferred to cell culture flask (Corning, USA) in Dulbecco’s Modified Eagle’s Medium supplemented with 10 % fetal bovine serum (FBS) (Biological Industries, Israel), 1 % Pen-Strep (Gibco, USA), and 1 % L-glutamine (Gibco, USA) and placed into the incubator at 37°C in a humidified incubator containing 5 % CO_2_. Cells from *passage 2* were harvested during the first expansion period.

### Cryopreservation

About 3 × 10^6^ AD-MSCs at *passage 2* were suspended in 1 ml of cryoprotectant solution containing 10 % dimethyl sulfoxide (Me^2^SO)(Sigma-Aldrich, USA)and 90 % FBS (Biological Industries, Israel).The cell suspension was kept in freezing tube (Corning, USA), cooled to − 80°C at a rate of − 1°C/min in freezing container (Nalgene, USA), and then transferred to nitrogen tank for long-term storage. At each freeze-thaw cycle, the stem cell characteristics of AD-MSCs were evaluated (Table 4).

**Table 4:** In-process test and cryopreservation criteria

Specification	Expected	Methods
Donor virology (RV, CDV, ICHV, CPV and CCV)	Negative	Elisa Kit
Viability	≥ 90 %	Trypan blue staining
Sterility	No growth	Microbiology culture
Mycoplasma	Negative	Culture
Endotoxin	> 0.5 EU/mL	Limulus Amebocyte Lysate
Phenotype (CD29, CD34, CD44, CD45 and CD90)	≥ 95 % CD29, CD34, CD90 ≤ 2 % CD34, CD45	Flow cytometry
Differentiation potential	Osteogenesis, adipogenesis	Induction culture

### Thawing and expansion

AD-MSCs was taken from nitrogen tank and rapidly thawed in water bath kettle of 37°C and transferred to a 15 ml centrifuge tube in 10×volume of PBS for washing twice. The thawed cells were cultivated and proliferated using the same protocol as described for primary expansion. AD-MSCs from *passage 3 to 7* were harvested for cytotherapy during this expansion period. All the cells for cytotherapy were evaluated synchronously (Table 5).

**Table 5:** Final product release criteria

Specification	Expected	Methods
Cell counts	1.0 × 10^6^ /kg	Automatic cell counter
Viability	≥ 90 %	Trypan blue staining
Sterility	No growth	Microbiology culture
Mycoplasma	Negative	Culture
Endotoxin	> 0.5 EU/mL	Limulus Amebocyte Lysate
Phenotype (CD29, CD34, CD44, CD45 and CD90)	≥ 95 % CD29, CD34, CD90 ≤ 2 % CD34, CD45	Flow cytometry
Differentiation potential	Osteogenesis, adipogenesis	Induction culture
Karyotype analysis	39 pairs of chromosomes	Giemsa staining
Tumorigenicity	Negative	Injection into nude mice

### Characterization and quality control

#### Cell counts and viability assessment

Accurate assessment of cell count and viability of AD-MSCs by the trypan blue dye exclusion test using automatic cell counter (Countess, Invitrogen, USA). The viability was calculated using the following formula: number of trypan blue-negative cells/number of total cell cells × 100.

#### Flow cytometry analysis

Cells were incubated with the following phycoerythrin (PE)-conjugated or fluorescein isothiocyanate (FITC)-conjugated antibodies: anti-CD29-PE (cat.no.303,004; BioLegend), anti-CD34-PE (cat.no.ab23830; Abcam), anti-CD44- FITC (cat.no.MA1-10229; Invitrogen), anti-CD45-FITC (cat.no.ab27287; Abcam), and anti-CD90-PE (cat.no.11-0900-81; Invitrogen) or their respective isotype controls. Cells were analyzed using a FACSCanto flow cytometry system (Beckman, USA). Data acquisition and analysis was performed with CytExpert.

#### In vitro differentiation assessment

In vitro adipogenic, osteogenic and chondrogenic differentiation were examined using MSCs Adipogenic Differentiation Kit (Cyanogen, China), Osteogenic Differentiation Kit and Chondrogenic Differentiation Kit (Cyanogen, China) following the manufacturer’s protocol for each kit. Adipogenic Differentiation Kit contains basic medium, fetal bovine serum, dexamethasone, insulin, IBMX and indomethacin. Osteogenic Differentiation Kit contains basic medium, fetal bovine serum, ascorbate, β-Glycerophosphate and dexamethasone. Chondrogenic Differentiation Kit contains basic medium, fetal bovine serum, ascorbate, dexamethasone, sodium Pyruvate, TGF-β3, insulin, transferrin and selenium. Cells were stained with Oil Red O solution to assess adipogenic differentiation, alizarin red solution to assess osteogenic differentiation and alcian blue solution to assess chondrogenic differentiation.

#### Population Doubling Time (Td) estimation

Cells were counted by automatic cell counter and cell population doubling time (Td) was calculated using the following formula: Td = t x lg2 / (lgNt - lgNo), where “No” refers to cell number after inoculation and “Nt” refers to cell number at T hour culture.

#### Colony-forming unit-fibroblasts estimation

AD-MSCs were seeded in 60-mm Petri dishes (100 cells/dish) cultured for 10 days, and stained with crystal violet solution. The number of colonies was determined under a camera, and clusters of more than 50 cells were considered colonies. The Colony-Forming unit-fibroblasts(CFU-F)was calculated using the following formula: CFU-F = colony number / initial cell number x 100 %.

#### Microbiology testing

Mycoplasma, Bacterium and fungus examination were performed in accordance with methods set forth in the Chinese Veterinary Pharmacopoeia. Sterility test was performed by inoculating samples in two different sterile nutrient mediums, namely, Fluid Thioglycolate Medium and Soybean Casein Digest Medium. Mycoplasma detection was done by inoculating samples in Mycoplasma Agar Medium and Mycoplasma Broth Medium.

#### Endotoxin testing

Endotoxin levels were determined by the gel clot limulus amebocyte lysate test in accordance with methods set forth in the Chinese Veterinary Pharmacopoeia.

#### Karyotype analysis

Chromosomes were prepared and banded by the G-banding technique according to standard methods. Following Giemsa staining, the numbers of chromosomes were calculated and analyzed.

#### Tumorigenicity assay

AD-MSCs and Hela (positive control) cells were delivered into the flank of 5-week-old female NOD/SCID mice, purchasing from Guangdong Experimental Animal Center. All mice were housed in a well-ventilated holding room with free access to food and water, and a 12/12 h light/dark cycle, with an ambient temperature of 20–25°C. After 40 days, tumors were excised after anesthesia with the injection of 3 % pentobarbital sodium (0.1 ml/100 g). Tumors were fixed by immersion in neutral buffered formalin and processed for standard hematoxylin and eosin staining.

### A case of cell therapy

A 2-month-old male golden retriever was diagnosed with leukopenia caused by canine parvovirus in February 2019. Clinical examination showed the body temperature rose to 39.2 degrees, the pulse rate was 110 beats/minute and the breathing was 30 beats/minute. Further blood test showed a significant decrease in white blood cells and neutrophils. It was finally diagnosed as CPV passing the CPV test strip. The case was treated with routine antiviral therapy and fluid supplementation while canine AD-MSCs were intravenously transplanted. The stem cells (1 × 10^6^ cells per kilogram of body weight) were diluted in normal saline containing 1 % canine serum albumin (Blood biotech, China) and transplanted into the sick dog once a day for 3 consecutive times. Biochemical indicators at 2, 3 days after treatment were tested to determine the treatment effect. Control group was another male golden retriever identified with parvovirus infection in the same litter. Clinical examination showed the body temperature rose to 38.5 degrees, the pulse rate was 110 beats/minute and the breathing was 22 beats/minute. The control case was treated with routine antiviral therapy and fluid supplementation.

### Statistical analysis

SPSS 17.0 software (SPSS, Inc.) was used for statistical analysis. Values are expressed as the means ± standard deviation. Statistical analysis was performed using one-way analysis of variance with the least-significant difference post hoc test. *P* < 0.05 was considered to indicate a statistically significant difference.

## Data Availability

The datasets used and/or analysed during the current study are available from the corresponding author upon reasonable request.

## Abbreviations

AD-MSCs: Adipose-derived Mesenchymal stem cells
CCV: Canine coronaviruses
CDV: Canine distemper virus
CFU-F: Colony-Forming unit-fibroblasts
CPV: Canine parvovirus
DMEM: Dulbecco's Modified Eagle's Medium
FBS: Fetal bovine serum
FITC: Fluorescein isothiocyanate
ICHV: Infectious canine hepatitis virus
Me^2^SO: Dimethyl sulfoxide
MSCs: Mesenchymal stem cells
PE: Phycoerythrin
RV: Rabies virus
Td: Population doubling time
